# Supplementary material for: Nitrogen Source Affects the Composition of Metabolites in Pepper (Capsicum annuum L.) and Regulates the Synthesis of Capsaicinoids through the GOGAT–GS Pathway
Source: Foods. 2020 Feb 5;9(2):150. doi: 10.3390/foods9020150 (PMC7073546; doi:10.3390/foods9020150)
Supplement: Supplementary file 1 [file foods-09-00150-s001.pdf]

Table S1. Significantly changed (VIP > 1 from partial least squares discriminant analysis and $p < 0.05$ from t-test) metabolites between pericarp and placenta.

Treatments	Peak	VIP	*p*	Fold change
T1 PE Vs PL	1-stearoyl-sn-glycero-3-phosphoethanolamine	1.18	0.00	3.79
	4-[4-(4-Hydroxy-3-methoxyphenyl)tetrahydro-1H,3H-furo[3,4-c]furan-1-yl]-2-methoxyphenyl hexopyranoside	1.02	0.03	15.24
	Glycerophospho-N-palmitoyl ethanolamine	1.06	0.00	3.65
	Dodecyl sulfate	1.44	0.00	3.59
	1-Caffeoyl-beta-D-glucose	1.25	0.03	0.01
	2-linoleoyl-sn-glycero-3-phosphoethanolamine	1.46	0.00	3.55
	Avenein	1.50	0.01	50.77
	Lariciresinol 4-O-glucoside	1.29	0.00	3.03
	Picrocrocin	1.29	0.02	1.94
	Sucrose	3.18	0.03	2.87
	2-[(11Z,14Z)-icosadienoyl]-sn-glycero-3-phosphoethanolamine	1.52	0.03	2.71
	Gentiopicrin	5.20	0.04	8.82
	L-(-)-Malic acid	7.36	0.02	2.36
	Gluconic acid	2.23	0.00	0.17
	Myristyl sulfate	1.42	0.00	2.82
T2 PE Vs PL	Î±-Lactose	1.28	0.00	3.14
	Melilotoside	1.36	0.01	0.06
	prostaglandin G2 2-glyceryl ester	1.33	0.00	7.34
	Asparagine	1.26	0.00	2.57
	4-Oxoproline	1.07	0.00	3.36
	Capsaicin	1.27	0.00	435.88
	Ascorbic acid	1.08	0.01	0.04
	D-(-)-Glutamine	1.03	0.05	4.22
	1-Caffeoyl-beta-D-glucose	1.22	0.00	0.01
	2-linoleoyl-sn-glycero-3-phosphoethanolamine	1.11	0.01	3.03
	13(S)-HpOTrE	1.68	0.00	114.88
	Lariciresinol 4-O-glucoside	1.04	0.00	3.22
	Dihydrocapsaicin	1.04	0.00	513.30
	Sucrose	5.06	0.00	6.69
	Fructosylglycine	1.06	0.00	7.98
	1-O-vanilloyl-beta-D-glucose	2.98	0.00	52.49
	Nonivamide	1.16	0.02	987.42
	L-Histidine	1.04	0.00	7.60
	Gentiopicrin	4.09	0.00	9.75
	Astragalin	2.85	0.02	0.05
	{2-[2-(Isobutyryloxy)-4-methylphenyl]-2-oxiranyl}methyl 2-methylbutanoate	1.25	0.00	999.94
	L-(-)-Malic acid	7.30	0.00	4.88

	Gluconic acid	1.28	0.00	0.18
	Î±-Lactose	1.36	0.02	2.93
	Melilotoside	1.74	0.00	0.05
	Asparagine	1.27	0.00	2.79
	4-Oxoproline	1.03	0.02	3.80
	Ascorbic acid	1.11	0.01	0.03
	D-(-)-Glutamine	1.21	0.02	6.05
	1-Caffeoyl-beta-D-glucose	1.13	0.00	0.01
T3	2-linoleoyl-sn-glycero-3-phosphoethanolamine	1.82	0.01	4.74
PE Vs PL	Lariciresinol 4-O-glucoside	1.15	0.00	2.75
	Fructosylglycine	1.10	0.01	8.46
	1-O-vanilloyl-beta-D-glucose	2.00	0.05	20.91
	L-Histidine	1.08	0.00	9.42
	Gentiopicrin	3.46	0.04	16.97
	Astragalin	2.20	0.00	0.10
	L-(-)-Malic acid	7.08	0.00	3.69
	Gluconic acid	1.99	0.00	0.07

Note: Peak, the material name from qualitative analysis; VIP, variable importance in the projection values from partial least squares discriminant analysis (PLS-DA); *P*, *p* value obtained by t-test; T1, NH_4^+:NO_3^- = 0:100; T2, NH_4^+:NO_3^- = 25:75; T3, NH_4^+:NO_3^- = 50:50; PE, pericarp; PL, placenta.

Table S2 Significantly changed (VIP > 1 from partial least squares discriminant analysis and $p < 0.05$) intercellular metabolites induced by N treatments.

Treatments	Tissue	Compounds	VIP	*P*	Fold change
T1 vs. T2	PE	Theophylline	4.97	0.023	1.68
		Ascorbic acid	1.81	0.029	2.44
		5-Aminolevulinic acid	1.08	0.024	0.29
		Gluconic acid	2.32	0.049	0.64
	PL	Î±-Lactose	1.37	0.00	2.25
		Prostaglandin G2 2-glyceryl ester	1.32	0.00	2.61
		Asparagine	1.37	0.00	2.06
		Capsaicin	1.36	0.02	5.44
		Theophylline	3.10	0.02	2.07
		N-Acetylvaline	1.02	0.00	2.72
		Avenein	1.16	0.03	0.21
		13(S)-HpOTrE	1.84	0.01	6.90
		Dihydrocapsaicin	1.11	0.01	5.63
		Sucrose	5.29	0.00	3.23
		Fructosylglycine	1.12	0.01	3.31
		1-O-vanilloyl-beta-D-glucose	2.81	0.02	2.71
		Nonivamide	1.32	0.03	17.42
		Cyclopentolate	1.11	0.04	13.08
		{2-[2-(Isobutyryloxy)-4-methylphenyl]-2-oxiranyl}methyl 2-methylbutanoate	1.20	0.04	3.08
T1 vs. T3	PE	Asparagine	1.14	0.029	0.36
		4-Oxoproline	1.43	0.026	0.36
		Theophylline	2.53	0.038	0.40
		Diethylpyrocarbonate	2.04	0.030	0.36
	PL	Î±-Lactose	1.10	0.04	2.06
		2-linoleoyl-sn-glycero-3-phosphoethanolamine	1.32	0.02	1.96
		Avenein	1.10	0.03	0.19
		D-(-)-Quinic acid	8.01	0.04	0.37
		Sucrose	5.91	0.04	4.50
		Thiolactomycin	7.75	0.04	0.39
T2 vs. T3	PE	Gentiopicrin	1.88	0.046	0.32
		Gluconic acid	1.79	0.023	1.50
	PL	4-(beta-D-glucosyloxy)benzoic acid	1.15	0.00	0.33
		Asparagine	1.14	0.04	0.77
		Dodecyl sulfate	1.12	0.02	0.60
		N-Acetylvaline	1.08	0.00	0.57
		13(S)-HpOTrE	2.37	0.01	0.17
		Dihydrocapsaicin	1.26	0.04	0.35

	1-O-vanilloyl-beta-D-glucose	3.59	0.02	0.35

Note: Peak, the material name from qualitative analysis; VIP, variable importance in the projection values from partial least squares discriminant analysis (PLS-DA); *P*, *p*- value obtained by t-test; T1, NH_4^+:NO_3^- = 0:100; T2, NH_4^+:NO_3^- = 25:75; T3, NH_4^+:NO_3^- = 50:50. PE, pericarp; PL, placenta.

Table S3. Parameter of the orthogonal projections to latent structures–discriminant analysis (OPLS-DA) model (Positive ion parameter).

Treatments	A	N	R^2X(cum)	R^2Y(cum)	Q^2(cum)
PE T1 Vs T2	1+2+0	6	0.929	0.964	0.778
PE T2 Vs T3	1+2+0	6	0.938	0.996	0.923
PE T1 Vs T3	1+2+0	6	0.952	0.997	0.986
PL T1 Vs T2	1+2+0	6	0.954	0.981	0.943
PL T2 Vs T3	1+3+0	6	0.943	1	0.992
PL T1 Vs T3	1+2+0	6	0.873	0.998	0.982
T1 PE Vs PL	1+2+0	6	0.888	0.998	0.99
T2 PE Vs PL	1+1+0	6	0.928	0.989	0.974
T3 PE Vs PL	1+1+0	6	0.897	0.968	0.869

Note: A, number of principal components of the model; N, number of observations of the model; R^2X (cum), the interpretability of the model to X variables; R^2Y (cum), the interpretability of the model to Y variable; Q^2 (cum), predictability of the model.
